# CAR-T cells targeting CD38 and LMP1 exhibit robust antitumour activity against NK/T cell lymphoma

**DOI:** 10.1186/s12916-023-03040-0

**Published:** 2023-08-30

**Authors:** Hongwen Li, Wenting Song, Jiazhuo Wu, Zhuangzhuang Shi, Yuyang Gao, Jiwei Li, Lijuan Han, Jianxiang Zhang, Zhaoming Li, Yong Li, Mingzhi Zhang

**Affiliations:** 1 Department of Oncology, Jianshendong Rd., The First Affiliated Hospital of Zhengzhou University, No. 1, Zhengzhou, 450052 Henan Province China; 2 State Key Laboratory of Esophageal Cancer Prevention and Treatment and Henan Key Laboratory for Esophageal Cancer Research, The First Affiliated Hospital of Zhengzhou University, Zhengzhou, Henan China; 3 Academy of Medical Sciences of Zhengzhou University, Zhengzhou, Henan China; 4 Department of Medicine, Baylor College of Medicine, Houston, TX USA

**Keywords:** CD38, LMP1, CAR-T, Tandem, Immunotherapy, NKTCL

## Abstract

**Background:**

Natural killer/T cell lymphoma (NKTCL) is an aggressive lymphoma with a poor prognosis. Chimeric antigen receptor-transduced T (CAR-T) cell therapy has become a promising immunotherapeutic strategy against haematologic malignancies.

**Methods:**

In this study, four CAR-T cell lines (CD38-CAR, LMP1-CAR, CD38-LMP1 tandem CAR 1 and CD38-LMP1 tandem CAR 2) were generated. The effect of CAR-T cells against NKTCL cells was evaluated both in vitro and in vivo. Expression of T cell activation markers and cytokines produced by CAR-T cells were detected by flow cytometry.

**Results:**

The four CAR-T cell lines could effectively eliminate malignant NKTCL cells. They could be activated and produce inflammatory cytokines in a target-dependent manner. In vivo tests showed that the CAR-T cells exhibited significant antitumour effects in a xenotransplanted NKTCL mouse model.

**Conclusions:**

In summary, four CAR-T cell lines exhibited significant cytotoxicity against NKTCL cells both in vitro and in vivo. These results indicated the effective therapeutic promise of CD38 and LMP1 CAR-T cells in NKTCL.

**Supplementary Information:**

The online version contains supplementary material available at 10.1186/s12916-023-03040-0.

## Background

NKTCL is an aggressive lymphoma associated with Epstein‒Barr virus (EBV) infection that predominantly occurs in Asia and South America [1–3]. NKTCL mainly involves extra-nodal sites and presents with localized symptoms, such as nasal obstruction and epistaxis, in the early stage. This disease may disseminate to multiple sites and cause general symptoms, including fever, weight loss and even haemophagocytic lymphohistiocytosis [4–7]. All types of NKTCL share the defining feature of EBV infection. Therefore, a positive result for EBV infection is essential for the diagnosis of NKTCL [8]. Chemotherapy combined with radiotherapy is the most common therapy for limited-stage NKTCL, with a 5-year overall survival (OS) of 72–74%. For patients with advanced-stage NKTCL, systemic chemotherapy is still the primary treatment. The dexamethasone, methotrexate, ifosfamide, l-asparaginase and etoposide (SMILE) regimen achieved prolonged OS and progression-free survival (PFS) compared with the conventional cyclophosphamide, hydroxydaunorubicin, oncovin and prednisone (CHOP) regimen [9]. In 2011, the novel chemotherapy dexamethasone, cisplatin, gemcitabine and pegaspargase (DDGP) regimen was developed and showed promising results in NKTCL patients [10].

Immunotherapy for NKTCL has developed slowly. Studies have shown that anti-CD30 and anti-PD1 antibodies may be effective therapeutic agents for relapsed/refractory NKTCL [11]. In the past decade, a promising and specific immunotherapy, CAR-T cell therapy, has drawn the attention of oncologists. CAR is a kind of synthetic receptor that fuses an antigen-recognizing domain with T cell signalling domains. T cells loaded with a CAR can recognize target molecules in an HLA-independent manner [12, 13]. CAR-T cells targeting CD19 are currently the most successful CAR-T cell therapy, which have shown efficacy and immunological specificity in the treatment of lymphomas and have been applied in the clinical administration of B cell malignancies [12, 14].

CD38 is involved in the pathogenesis and outcome of human immunodeficiency virus infection and chronic lymphocytic leukaemia [15]. Studies have shown the efficacy of treatments targeting CD38 molecules in multiple myeloma [16]. Latent membrane protein 1 (LMP1) is encoded by EBV. It has been demonstrated that LMP1 can induce malignant transformation in B cells and epithelial cells [17]. These results suggest the clinical potential of CD38- and LMP1-targeted therapies in CD38 + or LMP1 + malignancies.

In this study, we first explored the feasibility of CAR-T cell therapy targeting both CD38 and LMP1. Four CAR-T cell lines (CD38-CAR, LMP1-CAR, CD38-LMP1 tandem CAR 1 and CD38-LMP1 tandem CAR 2) were constructed, and their antitumour effects were evaluated both in vitro and in vivo.

## Methods

### Cell lines

The cell lines used in this study, NKYS, YT and KAI3, were obtained from Dr. Wing C. Chan (City of Hope Medical Center, Los Angeles, USA). Dr. Norio Shimizu and Yu Zhang of Chiba University provided SNK6 cells. SNT16 cells were a gift from Guangzhou Bairui Biomedical Technology Company, Ltd. NKYS, YT, KAI3 and SNT16 cells were cultured in RPMI-1640 medium supplemented with 10% FBS (Gibco, USA) and antibiotics (100 U/ml penicillin, 100 µg/ml streptomycin). Additional IL-2 (100 IU/ml) was added to the medium for NKYS and KAI3 cells. SNK6 cells were cultured in RPMI-1640 medium with 10% immune cell serum replacement (Gibco, USA), IL-2 (200 IU/ml) and antibiotics. All cells were cultured in an incubator (Thermo Fisher, USA) at 37 °C and 5% CO_2_. All cell lines tested negative for mycoplasma contamination, and cell-surface markers for these cell lines were validated by flow cytometry.

### Western blot analysis

Cells were lysed in NP40 lysis buffer and then centrifuged to harvest the protein supernatant. Twenty micrograms of proteins was resolved by SDS‒PAGE and transferred to the polyvinylidene fluoride membranes (Amersham Biosciences, USA). The membranes were blocked in Tris-buffered saline Tween buffer containing 5% (w/v) non-fat milk at room temperature for 1 h and then incubated with primary antibodies including anti-LMP1 (Abcam, ab78113) and anti-GAPDH (ProteinTech, 60,004–1-lg) at 4 °C overnight and then with secondary antibodies for 1 h at room temperature. Band images were digitally captured with a ChemiDocTM XRC + system (Bio-Rad Laboratories, USA).

### Immunofluorescence

NKYS, YT, KAI3, SNK6 and SNT16 cells were fixed in formalin and blocked in phosphate buffer solution (PBS) with 5% goat serum and 0.1% Triton X-100. Then, the cells were incubated overnight at 4 °C with an anti-LMP1 antibody (Abcam, ab78113). After washing 3 times with PBS, the cells were incubated with a Cy3-labelled goat-anti-mouse secondary antibody and DAPI. After washing with PBS, the cells were analysed under a fluorescence microscope (Nikon, Japan) at × 40 magnification.

### Immunohistochemistry

Tumour tissues of NKTCL patients or tumour-bearing mice were fixed in formalin, decalcified and paraffin-embedded. Following antigen retrieval, the sections were blocked with 0.3% H_2_O_2_ in methanol. The sections were boiled for 10 min in citrate buffer and blocked with serum-free protein block. Then, slides were incubated with anti-CD38 (Servicebio, GB114831), anti-LMP1 (Abcam, ab78113), anti-CD3ε (Servicebio, GB13014) or anti-CD56 (Servicebio, GB12041) primary antibodies and horseradish peroxidase (HRP)-labelled secondary antibody. For EBV-encoded RNA (EBER) in situ hybridization, the sections were treated with gastric enzymes and dehydrated with alcohol after dewaxing. Then, the sections were incubated with EBER probe and HRP-labelled anti-digoxin antibody. After incubation with HRP-labelled antibodies, the sections were washed three times with PBS and stained with DAB colour-developing solution. Then, the sections were counterstained with haematoxylin, dehydrated through a graded alcohol series, cleared in xylene and covered with coverslips. The percentage of positive cells was scored as follows: 0, negative expression (the frequency of positive cells was less than 5%); 1, weakly positive expression (the frequency of positive cells was 5% ~  < 25%); 2, positive expression (the frequency of positive cells ranged from 25 to < 50%); and 3, strongly positive expression (the frequency of positive cells was ≥ 50%).

### Lentiviral chimeric antigen receptor constructs and generation of lentiviral particles

The CAR sequences included the single-chain antibody variable region gene fragment (scFv) domain(s) of CD38- and/or LMP1-specific antibodies, a CD8a transmembrane domain, a 4-1BB costimulatory domain and a CD3ζ motif. The CAR sequences were synthetically produced (GENEWIZ, Suzhou, China) and cloned into the pEF-MCS-P2A-EGFP vector. Among these, the LMP1-CAR sequence was cloned and fused with or without EGFP. CD38-CAR and two Tan-CAR sequences were not fused with EGFP.

293 T cells were cultured in DMEM medium supplemented with 10% FBS and antibiotics. Then, pRSV-Rev, pLP-VSVG, pCMV-Gag-Pol vectors and CAR constructs (or vector) were transfected into 293 T cells using PEI transfection reagent (Sigma, USA). Seventy-two hours after transfection, cell-free supernatants were collected and concentrated using an ultrafiltration device (Merck Millipore, USA).

### Generation of CAR-T cells

Peripheral blood mononuclear cells (PBMCs) were isolated from the peripheral blood of healthy donors by Ficoll-Paque density centrifugation. Then, T cells were isolated from PBMCs using magnetic cell separation (MACS) and CD3 Dynabeads (Miltenyi Biotec, Germany) and cultured in X VIVO-15 medium (LONZA, Switzerland) supplemented with 200 IU/ml IL-2 and CD3/28 Dynabeads (Gibco, USA).

T cells were transduced by incubation with a CAR lentivirus (CAR-T) or vector lentivirus (Control T). After immediate centrifugation for 90 min at 37 °C, the transduced cells were cultured at 37 °C and 5% CO_2_ for 48 h. LMP1-CAR-EGFP fusion vector was used for the detection of transfection efficiency and in vitro cytotoxicity assay. LMP1-CAR vector without EGFP was used for the detection of T cell activation markers, cytokine production and in vivo experiments. The transfection efficiency of CD38-CAR or two Tan CAR-T cells was detected by fluorescence-activated cell sorter (FACS) using fluorescein isothiocyanate (FITC)-labelled recombinant human CD38 protein (ACROBiosystems, China).

### Cell-mediated cytotoxicity assay

Serial dilutions of CAR-T or control T cells (effector) were co-incubated with NKTCL cell lines (target) at E:T (effector-to-target) ratios of 4:1, 2:1, 1:1 and 1:2 for 6 h. The cytotoxicity of CAR-T cells was determined by measuring lactate dehydrogenase (LDH) release by impaired tumour cells. LDH in the supernatant was detected with a CytoTox 96® Non-Radioactive Cytotoxicity Assay kit (Promega, USA).

To evaluate the cytotoxicity of T cells at a lower E:T ratio, NKTCL cell lines were labelled with carboxyfluorescein diacetate and succinimidyl ester (CFSE) and then co-incubated with CAR-T or control T cells at an E:T ratio of 1:2 for 18 h. Then, the cells were stained with Annexin V and PI (Keygen Biotech, Jiangsu, China) and analysed on a FACSCalibur flow cytometer.

### Detection of T cell activation

CAR-T cells or control T cells were incubated with NKTCL cell lines (treatment group) at a ratio of 1:10 or with medium (blank group) for 24 h prior to detecting activation biomarkers of T cells. Then, different subsets of T cells were identified using fluorescein-conjugated antibodies specific for human CD4, CD8, CD25, CD69, CD38 and HLA-DR (BD Bioscience, USA).

### Cytokine measurements

A cytometric bead array (CBA) was used to detect the inflammatory cytokines and granzyme B in the cell supernatant. In brief, 2 × 10^4^ CAR-T or control T cells were co-cultured with 2 × 10^5^ target NKTCL cells (treatment group) or medium (blank group) in a 200-μl volume for 24 h. Then, 50 μl of cell supernatant or cytokine standard dilutions with different concentration was incubated with specific capture beads recognizing IL-2, IL-5, IL-6, IL-13, granulocyte–macrophage colony-stimulating factor (GM-CSF), granzyme B, interferon (IFN)-γ and tumour necrosis factor (TNF)-α and phycoerythrin (PE)-labelled detection antibodies (BD Bioscience, USA) for 2 h. The beads were washed, and the fluorescence intensity of capture beads was analysed by a standardized flow cytometry assay. The standard curve was calculated according to the average fluorescence intensity of the standard dilutions. Then, the concentration of cytokines in the cell supernatant was calculated according to the standard curve.

### In vivo experiment

The NSG mice (3–4 weeks old, female, 15–20 g) used in this study were obtained from the Shanghai Model Organisms Center (Shanghai, China). A total of 5 × 10^6^ NKYS-Luciferase cells were mixed with isopycnic Matrigel (Corning Incorporated, USA) and implanted subcutaneously in mice. The mice in treatment groups (*n* = 6 per group) received an intravenous tail vein injection of 4 CAR-T cells or control T cells on days 15 and 18 after tumour implantation, with a total of 1 × 10^7^ T cells infused per mouse (approximately 4 × 10^6^ transduced cells). Mice in the blank group were not treated. The tumour size was measured with a vernier calliper every 3 days for the first 50 days after implantation and daily after 50 days. The mice were given D-luciferin (150 mg/kg, i.p.) and anaesthetized with isoflurane. After 5 min, luminescence was detected using an in vivo imaging system (IVIS), and the intensity was quantitated and normalized with the Living Image software (PerkinElmer, MA, USA). A tumour size of 1000 mm^3^ or death of a mouse was considered an end event. After 75 days, the mice were euthanized by cervical dislocation.

### Statistical analysis

Statistical analysis was performed using GraphPad Prism version 5.0 (GraphPad Software, Inc., La Jolla, CA, USA). The results are reported as the mean ± standard deviation or median and interquartile range for repeated measurements. Analysis of variance (ANOVA) followed by the Bonferroni post-test was applied to assess the differences among normally distributed samples, and the Mann‒Whitney test was used for non-normally distributed samples. A value of *P* < 0.05 was considered statistically significant.

## Results

### Expression of CD38 and LMP1 in NKTCL patients and cell lines

**Fig. 1:**
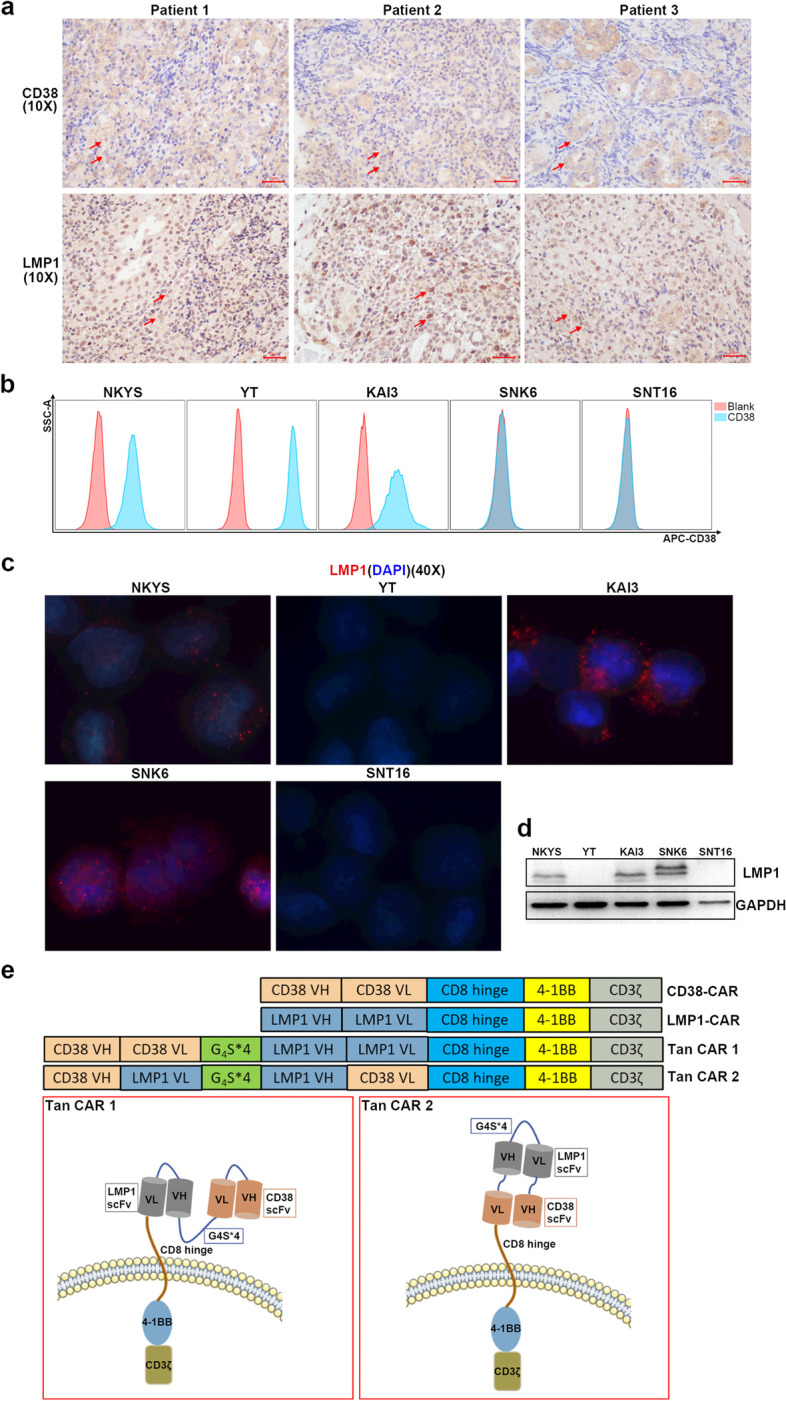
CD38 and LMP1 expression in NKTCL patients and cell lines. **a** CD38 and LMP1 expression in NKTCL patients. Arrows showed the positive cells. Scale bar: 10 μm. **b** CD38 expression in NKTCL cell lines detected by FACS. **c** LMP1 expression in NKTCL cell lines detected by immunofluorescence. Red fluorescence showed the positive cells. **d** LMP1 expression in NKTCL cell lines detected by western blot. **e** Fragment sequences of four CARs and constructs of two Tan CARs

The expression of CD38 and LMP1 in tumour tissues from 10 NKTCL patients was evaluated (Fig. 1a, Additional file 1: Fig. S1). A total of 7/10 patients were positive for CD38 (score over 1), and 5/10 patients were positive for LMP1 (score over 1) (Table 1).

**Table 1:** CD38 and LMP1 expression in NKTCL

	Number of cases	Positivity score			
		3	2	1	0
CD38	10	4	3	1	2
LMP1	10	2	3	3	2

The NKTCL cell lines NKYS, YT, KAI3, SNK6 and SNT16 were used as target cells in this study. FACS detection showed homogeneous expression of CD38 on NKYS, YT and KAI3 cells and negative expression on SNK6 and SNT16 cells (Fig. 1b). Immunofluorescence and western blot analyses showed positive expression of LMP1 in NKYS, KAI3 and SNK6 cells (Fig. 1c, d). The 5 target cell lines could be divided into four groups: CD38 + LMP1 + cells (NKYS and KAI3), CD38 + LMP1 − cells (YT), LMP1 + CD38 − cells (SNK6) and CD38-LMP1 − cells (SNT16).

### Generation of CAR-T cells

The anti-CD38 and anti-LMP1 scFv sequences were based on published CD38- and LMP1-specific antibody sequences [18, 19]. Two single-targeted CARs (CD38-CAR and LMP1-CAR) contained the corresponding scFv domain, while two tandem CARs (Tan CAR 1 and Tan CAR 2) contained both scFv domains in different orders connected via a G4S linker (Fig. 1e). The CD38-CAR or two Tan CAR-T cells could be labelled by recombinant human CD38 protein and detected by FACS, which suggested the binding affinity to CD38 protein of CAR-T cells. The affinity of LMP1-CAR and two Tan CAR-T cells to LMP1 protein was demonstrated by their different effects on LMP1 + or LMP1 − tumour cells in subsequent in vitro and in vivo experiments (Additional file 1: Fig. S2).

### Target-dependent cytotoxicity of CAR T cells to tumour cells

**Fig. 2:**
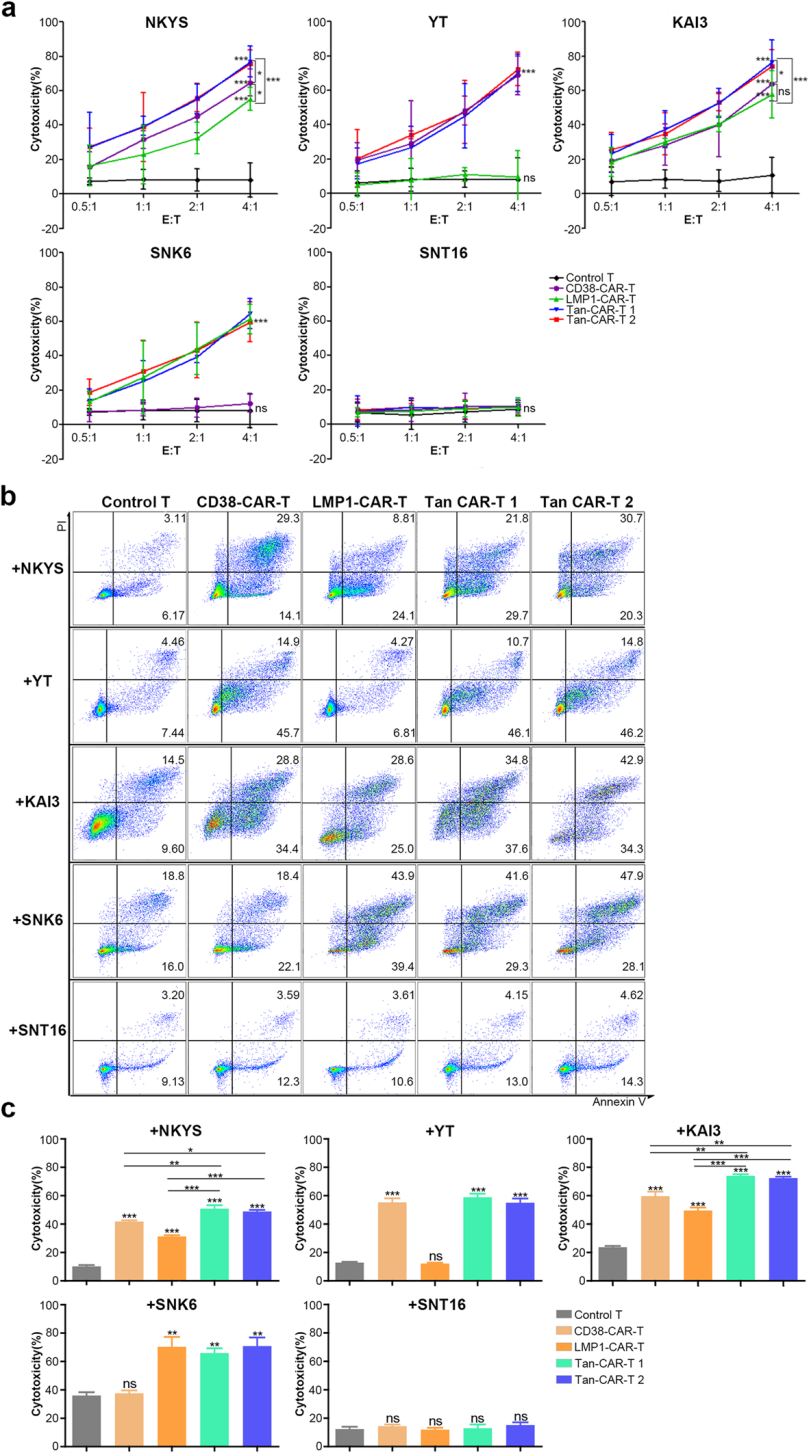
Cytotoxicity of CAR-T cells. **a** CAR-T or control T cells were co-incubated with NKTCL cell lines for 6 h. The cytotoxic effect of CD38-CAR-T and control T cells were evaluated by detecting LDH released by tumour cells. **b** CAR-T or control T cells were co-incubated with CFSE-labelled NKTCL cell lines for 18 h. Cells were stained with Annexin V and PI to evaluate the cytotoxicity of T cells. A value of *p* < 0.05 was considered statistically significant. ns, not significant; *p < 0.05; **p < 0.01; ***p < 0.001

LDH released by lysed tumour cells was detected to evaluate the cytotoxicity of CAR-T cells. Compared with control T cells, CD38-CAR and LMP1-CAR-T cells showed significantly higher cytotoxicity towards CD38 + target cells (NKYS, YT and KAI3) and LMP1 + target cells (NKYS, KAI3 and SNK6), respectively, at an E:T ratio of 4:1. However, there was no difference in CD38 − (SNK6 and SNT16) or LMP1 − (YT and SNT16) cells. Tan CAR 1 and Tan CAR 2 T cells efficiently lysed both CD38 + and LMP1 + target cells (NKYS, YT, KAI3 and SNK6). However, they showed no apparent cytotoxicity towards CD38-LMP1 − cells (SNT16). The response of control T cells to all target NKTCL cells was limited. We also analysed the cytolytic activity of single-targeted and Tan CAR-T cells. The two Tan CAR T cells showed significantly higher cytotoxicity to CD38 + LMP1 + cells (NKYS and KAI3) than CD38-CAR and LMP1-CAR-T cells (Fig. 2a).

For the determination of the antitumour effect at a lower E:T ratio, CAR-T cells were co-incubated with NKTCL cell lines labelled with CFSE for 18 h. At an E:T ratio of 1:2, CAR-T cells showed significant cytotoxicity against target-positive NKTCL cells (Fig. 2b, c). Similar to previous results, the 2 Tan CAR-T cells showed significantly higher cytotoxicity to CD38 + LMP1 + cells than 2 single-targeted CAR-T cells (Fig. 2b, c).

### Activation and cytokine secretion of CAR-T cells

**Fig. 3:**
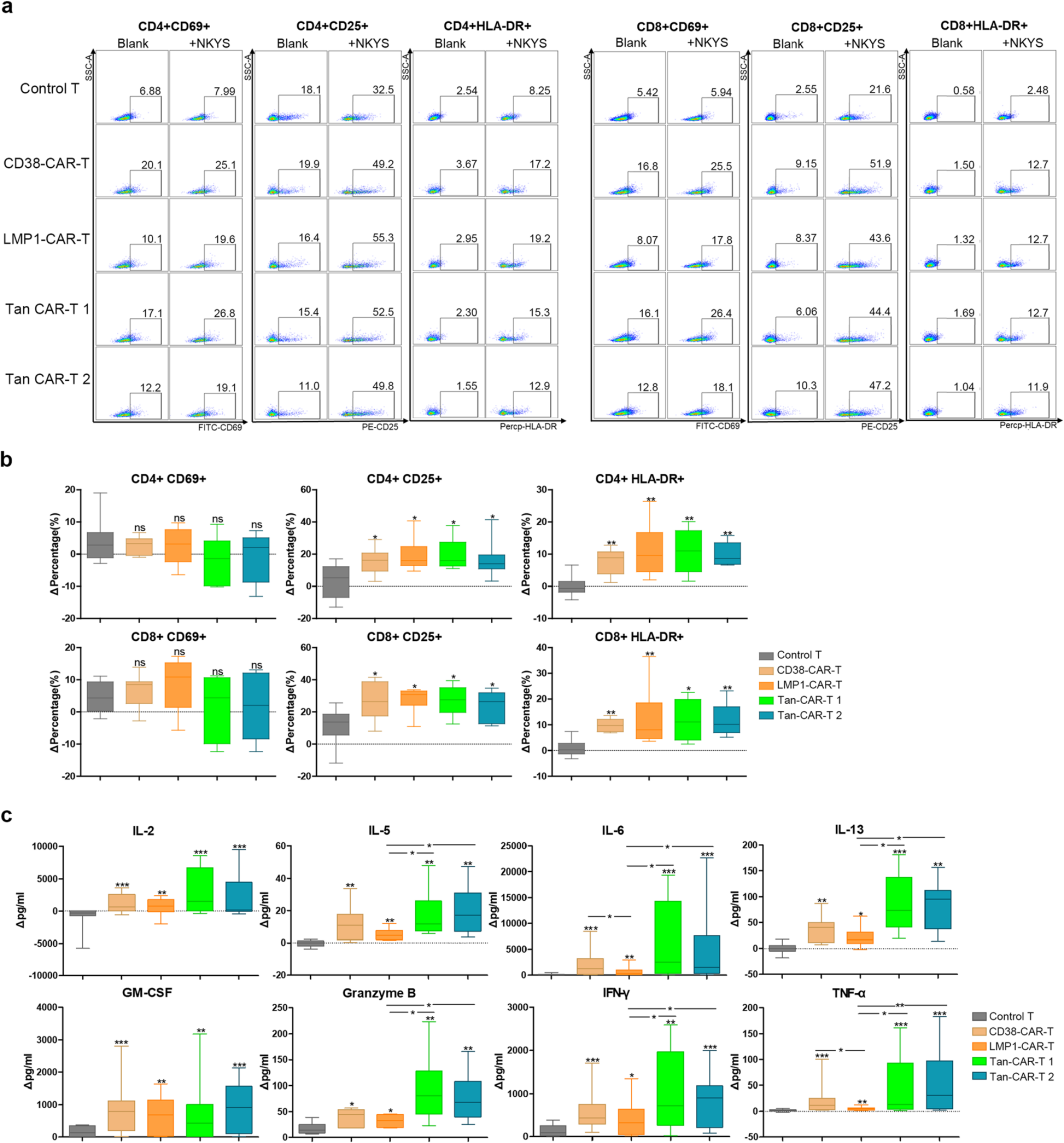
Activation and cytokines releasing of CAR-T cells co-cultured with NKYS. **a** Activation markers (CD69, CD25 and HLA-DR) expression on CD4 + and CD8 + subgroups of T cells were detected by FACS. **b** Statistical results of activation markers expression on T cells. ΔPercentage is equal to the difference between the proportion of positive subpopulation of T cells in the treatment group and blank group. **c** Cytokines released by CAR-T cells and control T cells were detected using the CBA Kit. Δpg/ml refers to the difference between cytokine concentration in the treatment and blank groups. A value of *p* < 0.05 was considered statistically significant. ns, not significant; *p < 0.05; **p < 0.01; ***p < 0.001

Stimulation of T cells may lead to the upregulation of surface cellular activation markers, including early (CD69) and late (CD25 and HLA-DR) activation markers [20]. To evaluate the target-dependent activation of T cells, control T and CAR-T cells were co-cultured with all 5 target NKTCL cell lines for 24 h. The data showed that co-incubation with target-positive NKTCL cells could significantly increase the expression of CD25 and HLA-DR in both the CD4 + and CD8 + subgroups of CAR-T cells (Fig. 3a, b, Additional file 1: Figs. S3-S4).

Inflammatory cytokines and granzyme B released by T cells were detected. After 24 h of co-culturing the 4 CAR-T cells or control T cells with target NKTCL cells, the cell supernatant was harvested, and the concentrations of IL-2, IL-5, IL-6, IL-13, GM-CSF, granzyme B, IFN-γ and TNF-α were measured by flow cytometry. The data showed that the 4 CAR-T cell lines stimulated by target-positive NKTCL cells generated significantly increased levels of these cytokines compared with control T cells (Fig. 3c, Additional file 1: Fig. S5-S6). When co-cultured with CD38 + LMP1 + NKYS cells, the two Tan CAR-T cell lines produced more IL-5, IL-6, IL-13, granzyme B, IFN-γ and TNF-α than LMP1-CAR-T cells. Similar results were also observed for the other CD38 + LMP1 + cell line, KAI3.

### *Effectiveness of CAR-T cells in the treatment of NKTCL *in vivo

**Fig. 4:**
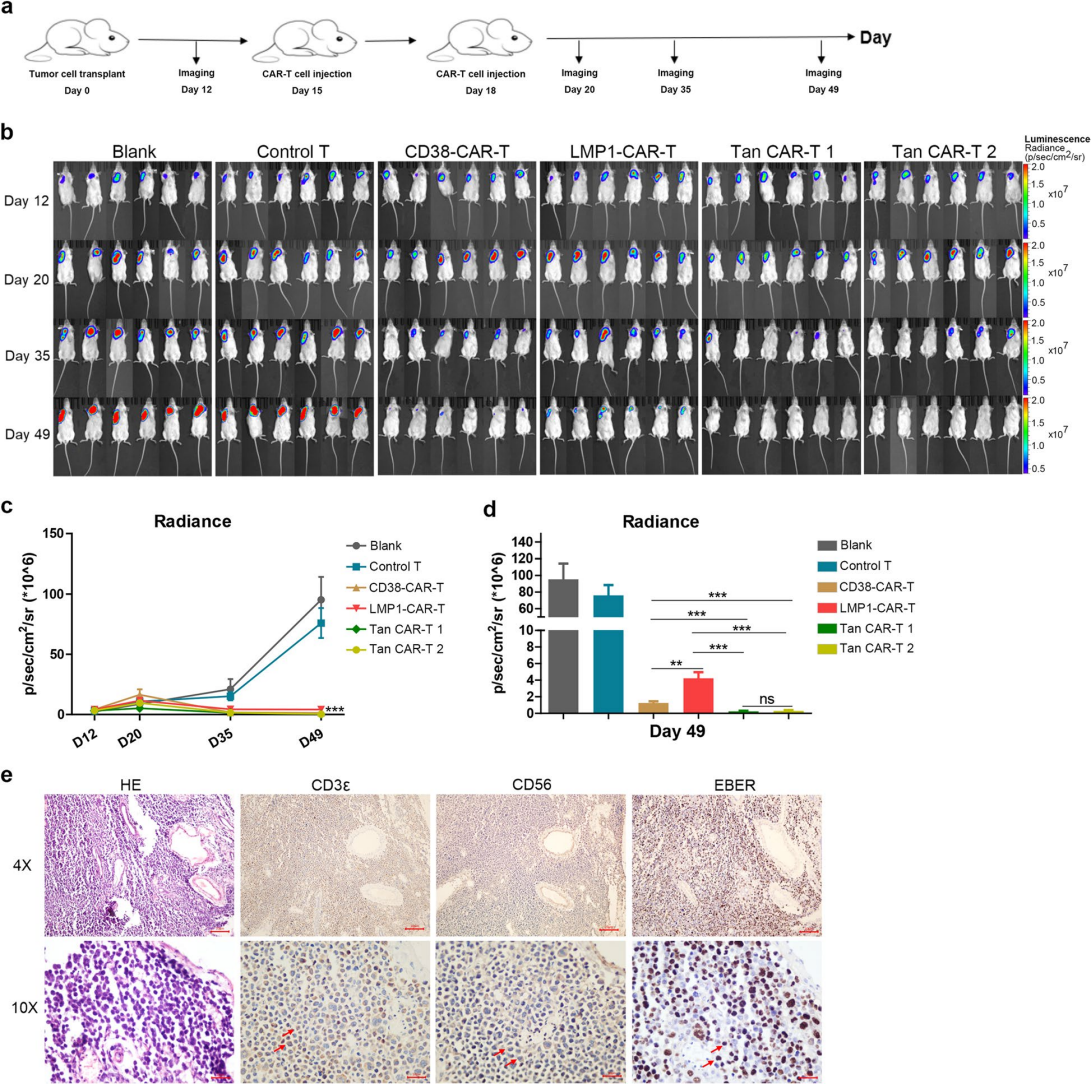
Effectiveness of CAR-T cells on NKTCL in vivo. **a** Overview of the treatment of xenograft tumour model in vivo. **b**, **c** Tumour growth was monitored by IVIS living imaging. The mouse tumour burden was indicated by bioluminescence radiance. **d** Tan CAR-T cells showed a significant anti-tumour effect compared to CD38- or LMP1-CAR-T cells. **e** IHC showed that tumours isolated from mice were positive for CD3ε, CD56 and EBER. Arrows showed the positive cells. Scale bar: 10 μm. A value of *p* < 0.05 was considered statistically significant. ns, not significant; *p < 0.05; **p < 0.01; ***p < 0.001

The in vitro studies demonstrated that the 4 CAR-T cell lines exhibited valid antitumour effects compared with control T cells. Hence, we further investigated the efficacy of CAR-T cells against NKTCL cells in vivo in a subcutaneous NKYS tumour xenograft NSG mouse model. The tumour burden was monitored by measuring the luminescence intensity using IVIS. Twelve days after tumour cell implantation, mice were treated with an intravenous injection of control T or CAR-T cells (Fig. 4a). Tumours showed fast progression in the untreated group and control T cell-treated group (Fig. 4b). Treatment with CAR-T cells induced a significant antitumour effect (Fig. 4c). The data also showed smaller tumour burdens in the two groups of mice treated with Tan CAR-T cells compared with those treated with single-target CAR T cells, but there were no significant differences among two Tan CAR-T cell-treated groups (Fig. 4d). CAR-T cell therapy led to a significant survival advantage compared with the blank and control groups (Additional file 1: Fig. S7). Tumours isolated from mice were stained positive for CD3ε, CD56 and EBER via immunohistochemistry (Fig. 4e).

## Discussion

In the last decade, CAR-T cell therapy has shown tremendous potential in the treatment of B cell leukaemia and lymphoma [21, 22]. Our previous study showed that the administration of CAR-T cells is a promising immunotherapeutic strategy for T-lymphoblastic leukaemia/lymphoma [23]. To date, studies on CAR-T cell therapy for NKTCL are limited. CAR-T cells targeting CD38 and B7-H3 have been evaluated as two immunotherapeutic agents for NKTCL therapy [24, 25].

CD38 is a type II transmembrane glycoprotein expressed in a variety of immune cells, including T cells, B cells, NK cells, macrophages and dendritic cells [26]. CD38 has been developed as a promising target for malignancy treatment. A clinical trial (NCT02927925) showed that daratumumab, an anti-CD38 monoclonal antibody, achieved an overall response rate (ORR) of 25.0% in the treatment of relapsed or refractory NKTCL [27]. Researchers have also evaluated the feasibility of CD38-CAR-T cell therapy in a variety of lymphoid neoplasms, including multiple myeloma and NKTCL [18, 21, 25].

EBV is observed in almost all NKTCL tumour cells [28]. LMP1 is an integral transmembrane protein encoded by EBV and may be related to increased malignant neoplasia and a decrease in the immune response [29]. LMP1 may induce malignant transformation in B cells and epithelial cells by inducing cell surface adhesion molecules, activating antigens and upregulating antiapoptotic proteins [17, 30–32].

In this study, we evaluated the expression of CD38 and LMP1 in NKTCL patients. The data showed that CD38 was highly expressed (7/10 positive) in NKTCL tumour tissues. Although LMP1 was positive in only 5/10 NKTCL patients, its role in malignant transformation and the immune response made it a non-negligible molecule. These results indicated that these proteins could be potential targets for immunotherapy in NKTCL. We constructed two single-targeted CARs, CD38-CAR and LMP1-CAR. Both CAR-T cell lines could be activated and release increased amounts of cytokines when incubated with CD38- or LMP1-positive NKTCL cells. When co-cultured with NKTCL cell lines, CAR-T cells showed increased expression of late T cell activation markers (CD25 and HLA-DR) in both the CD4 + and CD8 + subgroups. When activated by target antigens, CAR-T cells can release cytokines and lyse tumour cells [33]. In our study, CAR-T cells stimulated by NKTCL cells generated relatively high levels of IL-2, IL-5, IL-6, IL-13, GM-CSF, granzyme B, IFN-γ and TNF-α. The production of IL-2, IL-6 and IFN-γ showed the greatest changes. IL-2 promotes the proliferation of immunocytes and the transcription of antiapoptotic proteins [34]. IFN-γ is involved in anti-proliferative, anti-angiogenic and pro-apoptotic effects against malignant cells [35]. IL-6 is involved in chronic autoimmune inflammation and can induce cytokine storm. Treatment with tocilizumab, an anti-IL-6 receptor antibody, has been found to alleviate cytokine storm induced by chimeric antigen receptor (CAR)-T cell therapy [36].

Our data also showed significant cytolytic activity of CD38 − and LMP1-CAR-T cells against NKTCL cells both in vitro and in vivo. Compared with control T cells, CAR-T cells showed significantly higher cytotoxicity to NKTCL cell lines. The LDH assay and Annexin V assay indicated that the two single-targeted CAR-T cells could eliminate CD38 + or LMP1 + NKTCL cells rapidly and efficiently. However, they showed no effect on CD38 − or LMP1 − cell lines. These results indicate that CAR-T cells can recognize the corresponding target molecules and eliminate tumour cells in an HLA-dependent manner. The in vitro cytotoxicity of CAR-T cells was supported by the results generated with the in vivo mouse model. Tumours progressed rapidly in mice not given CAR-T cell treatment or given control T cell infusion. Treatment with CAR-T cells significantly inhibited tumour growth.

Due to the variable antigenic heterogeneity in tumour cells, CAR-T cell therapies targeting more than one antigen are of interest to researchers. A Tan CAR includes two antigen-binding domains in a single CAR structure. Tan CAR-T cells are capable of lysing target cells upon loss of one of the target molecules and produce a synergistic enhancement effect by recognizing both targets [37, 38]. Tumours that have relapsed or become refractory due to antigen escape are one of the most common challenges in targeted therapy. Therapy with Tan CAR-T cells specific for two distinct targets may be a feasible approach to overcome antigen escape and broaden the specificities of CAR T cells. The presence of scFvs against two target antigens could enhance T cell activation and cytolysis through an increased avidity [37]. To date, several Tan CAR T cells have undergone preclinical evaluation against malignant cell lines, such as CD19-CD20, CD38-BCMA and CD70-B7-H3 Tan CAR-T cells [38–40].

We constructed two Tan CARs. Both Tan CAR-T cell lines could be activated by CD38- and/or LMP1-positive NKTCL cells. Increased cytokine release also demonstrated the target-dependent activation of the Tan CAR-T cells. Following stimulation with CD38 + LMP1 + cells (NKYS and KAI3), the two Tan CAR-T cell lines produced more IL-13, granzyme B, IFN-γ and TNF-α than LMP1-CAR-T cells, which suggested that the cytokine production of Tan CAR-T cells was mainly affected by the recognition of CD38. They showed stronger cytolytic activity against tumour cells than single-targeted CAR-T cells both in vitro and in vivo. Compared to the two single-targeted CAR-T cell lines, the two Tan CAR-T cell lines produced similar effects against YT (CD38 + LMP1 −) and SNK6 (CD38-LMP1 +) cells. These results verified that Tan CARs produce a synergistic enhancement effect via recognition of both targets and cytolytic activity towards NKTCL cells upon loss of one of the target molecules.

## Conclusion

In this study, for the first time, both CD38 and LMP1 were used as immunotherapeutic targets in NKTCL. All four CAR-T cell lines exhibited cytotoxicity against NKTCL cells in vitro and in vivo. These CAR-T cells may provide a promising and effective solution for the treatment of lymphomas. In particular, the two Tan CARs may avoid the challenge of antigen escape during immunotherapy. They may provide a promising and effective solution for the treatment of NKTCL in the future.

### Supplementary Information

**Additional file 1:  Fig. S1.** A: CD38 and LMP1 expression in NKTCL patients. **Fig. S2.** CAR expression was detected by FITC-labeled human CD38 protein or EGFP. **Fig. S3.** Activation markers (CD69, CD25 and HLA-DR) expression on CD4+ and CD8+ subgroups of CAR-T cells co-cultured with YT, KAI3, SNK6 or SNT16. **Fig. S4.** Statistical results of activation markers expression on CAR-T cells co-cultured with YT, KAI3, SNK6 or SNT16. **Fig. S5.** Statistical results of cytokines releasing of CAR-T cells co-cultured with YT, KAI3, SNK6 or SNT16. **Fig. S6.** Flow histogram showed fluorescence intensity of each cytokine. **Fig. S7.** Survival curve data for each experimental group.

## Data Availability

The datasets used and analysed during the current study are available from the corresponding author upon reasonable request.

## Abbreviations

ANOVA: Analysis of variance
CAR-T: Chimeric antigen receptor-transduced T
CBA: Cytometric bead array
CFSE: Carboxyfluorescein diacetate, succinimidyl ester
CHOP: Cyclophosphamide, hydroxydaunorubicin, oncovin and prednisone
DDGP: Dexamethasone, cisplatin, gemcitabine and pegaspargase
EBER: EBV-encoded RNA
EBV: Epstein‒Barr virus
FACS: Fluorescence-activated cell sorter
FITC: Fluorescein isothiocyanate
GM-CSF: Granulocyte-macrophage colony-stimulating factor
HRP: Horseradish peroxidase
IFN: Interferon
IVIS: In vivo imaging system
LDH: Lactate dehydrogenase
LMP1: Latent membrane protein 1
MACS: Magnetic cell separation
NKTCL: Natural killer/T cell lymphoma
ORR: Overall response rate
OS: Overall survival
PBMCs: Peripheral blood mononuclear cells
PBS: Phosphate buffer solution
PE: Phycoerythrin
PFS: Progression-free survival
scFv: Single-chain antibody variable region gene fragment
SMILE: Dexamethasone, methotrexate, ifosfamide, l-asparaginase and etoposide
TNF: Tumour necrosis factor
