# Supplementary material for: CAR-T cells targeting CD38 and LMP1 exhibit robust antitumour activity against NK/T cell lymphoma
Source: BMC Med. 2023 Aug 30;21:330. doi: 10.1186/s12916-023-03040-0 (PMC10470138; doi:10.1186/s12916-023-03040-0)
Supplement: Supplementary file 1 — Additional file 1: Fig. S1. A: CD38 and LMP1 expression in NKTCL patients. Fig. S2. CAR expression was detected by FITC-labeled human CD38 protein or EGFP. Fig. S3. Activation markers (CD69, CD25 and HLA-DR) expression on CD4+ and CD8+ subgroups of CAR-T cells co-cultured with YT, KAI3, SNK6 or SNT16. Fig. S4. Statistical results of activation markers expression on CAR-T cells co-cultured with YT, KAI3, SNK6 or SNT16. Fig. S5. Statistical results of cytokines releasing of CAR-T cells co-cultured with YT, KAI3, SNK6 or SNT16. Fig. S6. Flow histogram showed fluorescence intensity of each cytokine. Fig. S7. Survival curve data for each experimental group. [file 12916_2023_3040_MOESM1_ESM.zip › fig s2R5.pdf]

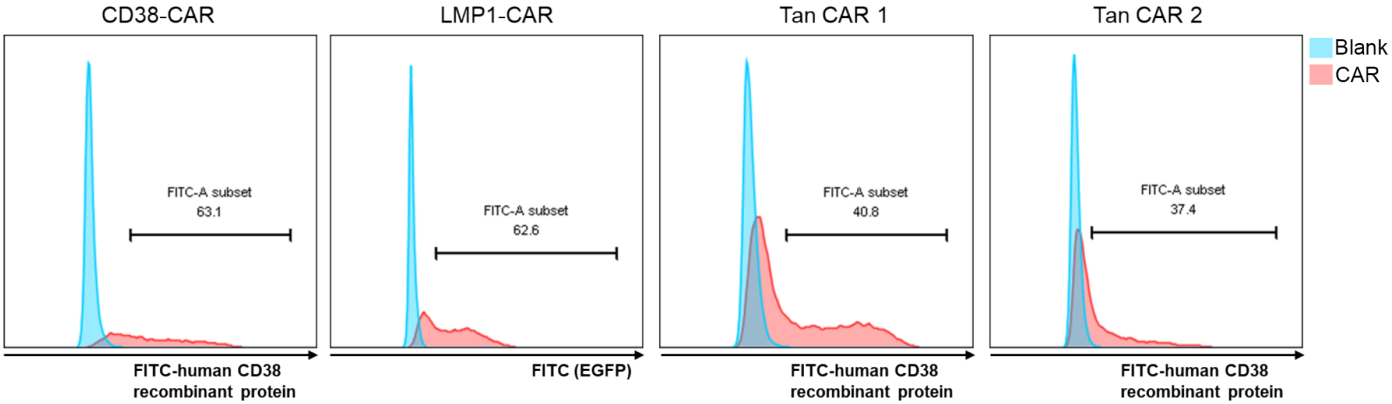

CD38-CAR
LMP1-CAR
Tan CAR 1
Tan CAR 2
Blank
CAR
FITC-A subset
63.1
FITC-A subset
62.6
FITC-A subset
40.8
FITC-A subset
37.4
FITC-human CD38 recombinant protein
FITC (EGFP)
FITC-human CD38 recombinant protein
FITC-human CD38 recombinant protein
