# Supplementary figures and images for: CAR-T cells targeting CD38 and LMP1 exhibit robust antitumour activity against NK/T cell lymphoma
Source: BMC Med. 2023 Aug 30;21:330. doi: 10.1186/s12916-023-03040-0 (PMC10470138; doi:10.1186/s12916-023-03040-0)

Patient 4

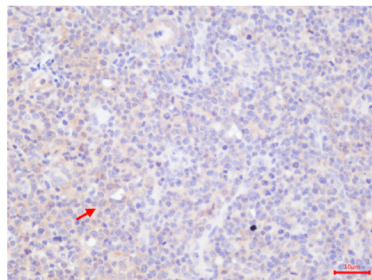

Patient 5

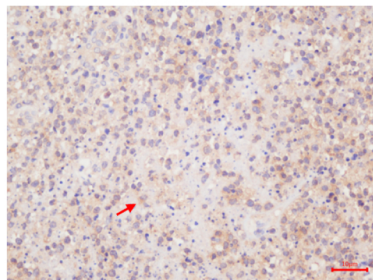

Patient 6

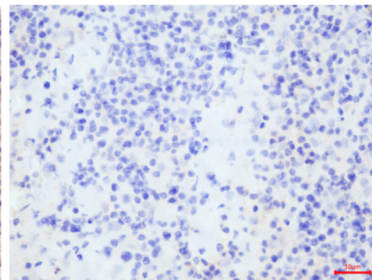

Patient 7

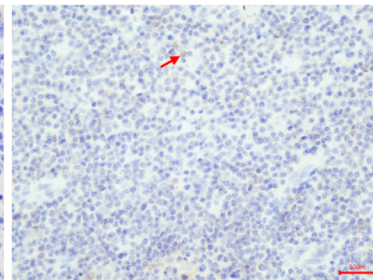CD38  
(10X)LMP1  
(10X)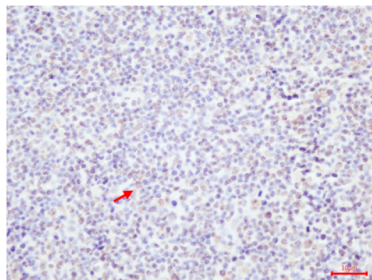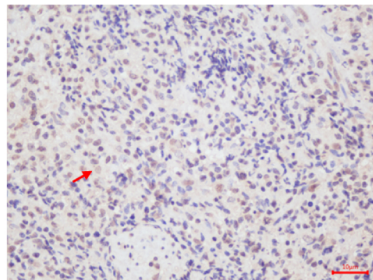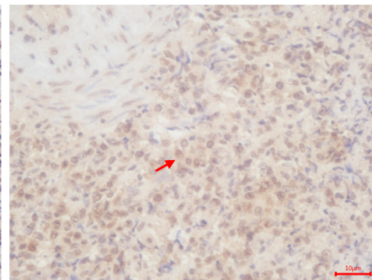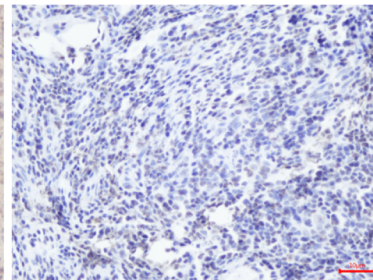

Patient 8

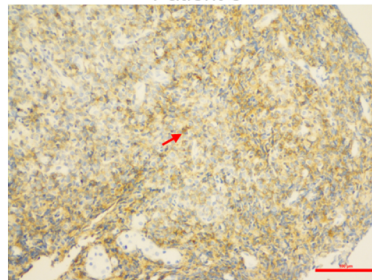

Patient 9

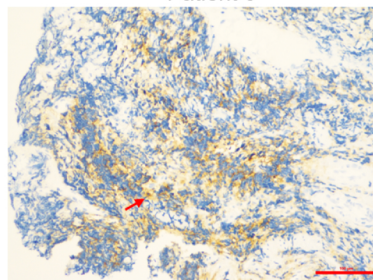

Patient 10

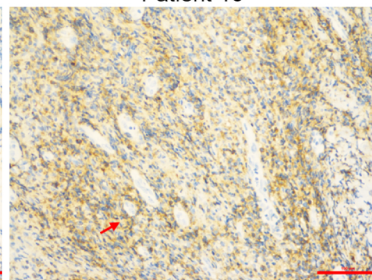CD38  
(10X)LMP1  
(10X)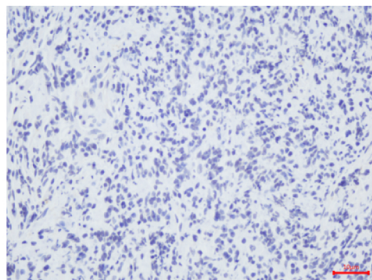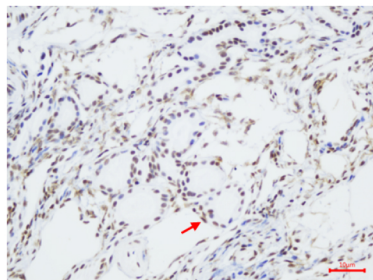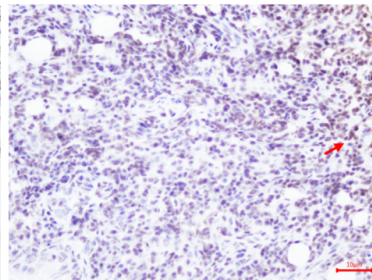

Supplement: Supplementary file 1 — Additional file 1: Fig. S1. A: CD38 and LMP1 expression in NKTCL patients. Fig. S2. CAR expression was detected by FITC-labeled human CD38 protein or EGFP. Fig. S3. Activation markers (CD69, CD25 and HLA-DR) expression on CD4+ and CD8+ subgroups of CAR-T cells co-cultured with YT, KAI3, SNK6 or SNT16. Fig. S4. Statistical results of activation markers expression on CAR-T cells co-cultured with YT, KAI3, SNK6 or SNT16. Fig. S5. Statistical results of cytokines releasing of CAR-T cells co-cultured with YT, KAI3, SNK6 or SNT16. Fig. S6. Flow histogram showed fluorescence intensity of each cytokine. Fig. S7. Survival curve data for each experimental group. [file 12916_2023_3040_MOESM1_ESM.zip › fig s1R5.pdf]

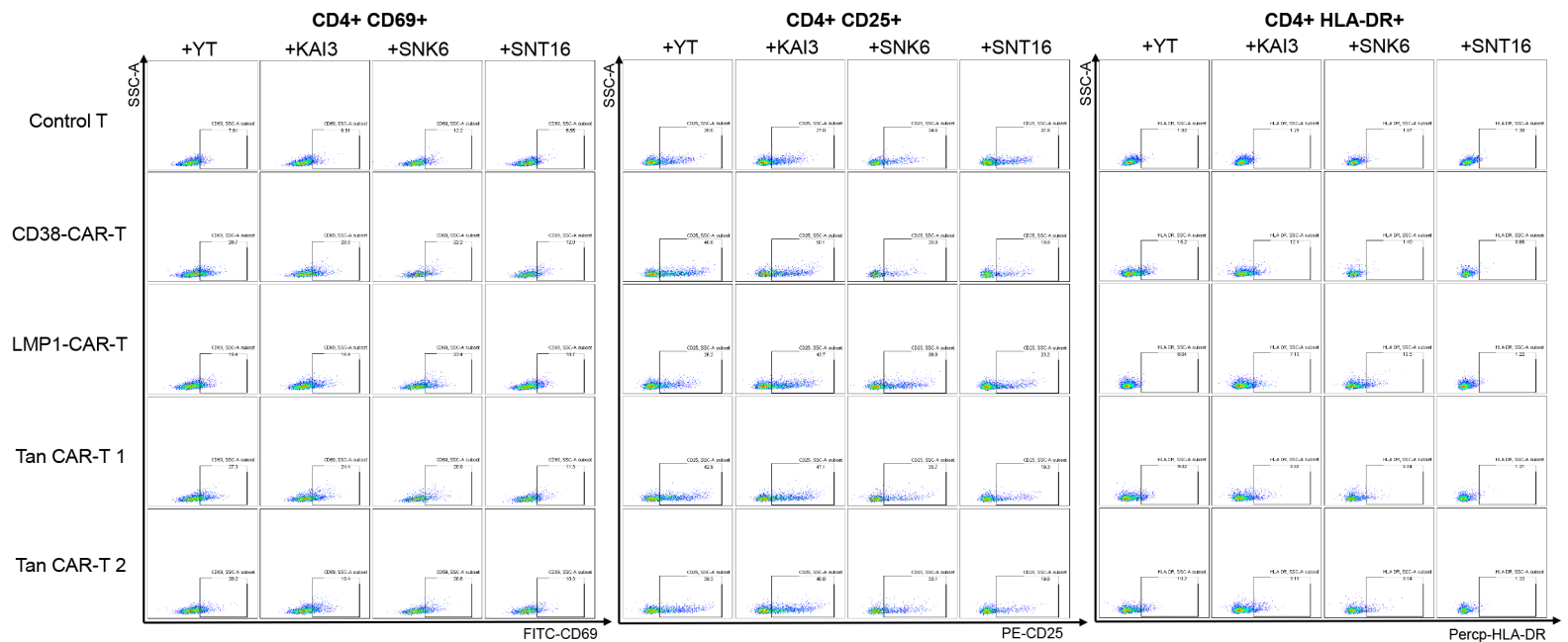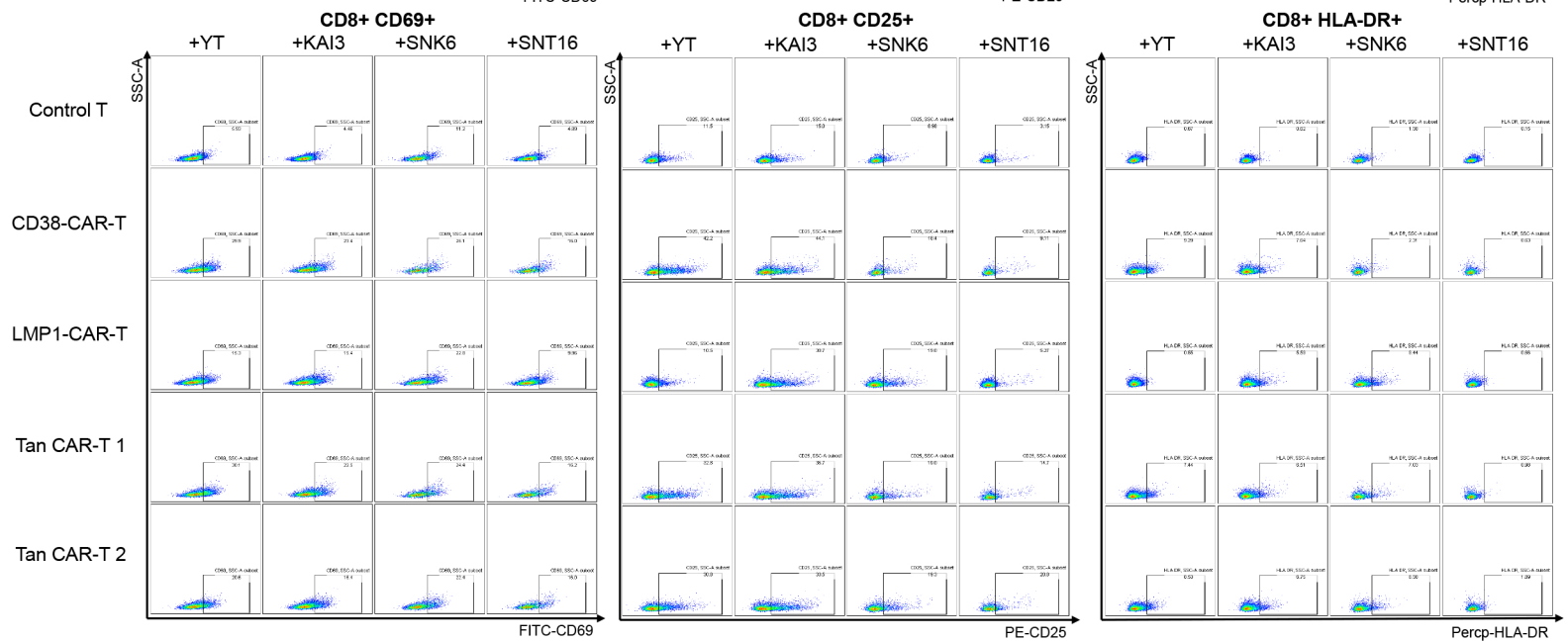

Supplement: Supplementary file 1 — Additional file 1: Fig. S1. A: CD38 and LMP1 expression in NKTCL patients. Fig. S2. CAR expression was detected by FITC-labeled human CD38 protein or EGFP. Fig. S3. Activation markers (CD69, CD25 and HLA-DR) expression on CD4+ and CD8+ subgroups of CAR-T cells co-cultured with YT, KAI3, SNK6 or SNT16. Fig. S4. Statistical results of activation markers expression on CAR-T cells co-cultured with YT, KAI3, SNK6 or SNT16. Fig. S5. Statistical results of cytokines releasing of CAR-T cells co-cultured with YT, KAI3, SNK6 or SNT16. Fig. S6. Flow histogram showed fluorescence intensity of each cytokine. Fig. S7. Survival curve data for each experimental group. [file 12916_2023_3040_MOESM1_ESM.zip › fig s3R5.pdf]

+YT

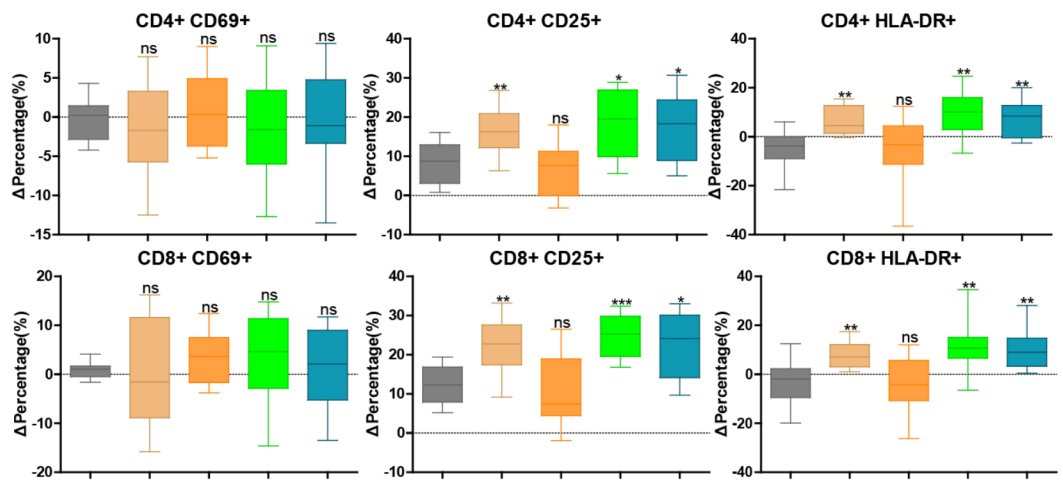

+KAI3

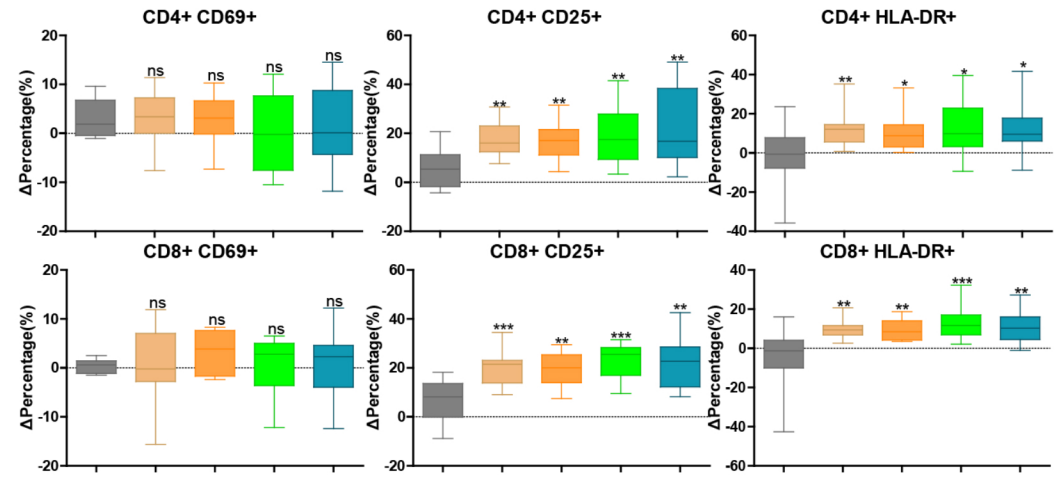

+SNK6

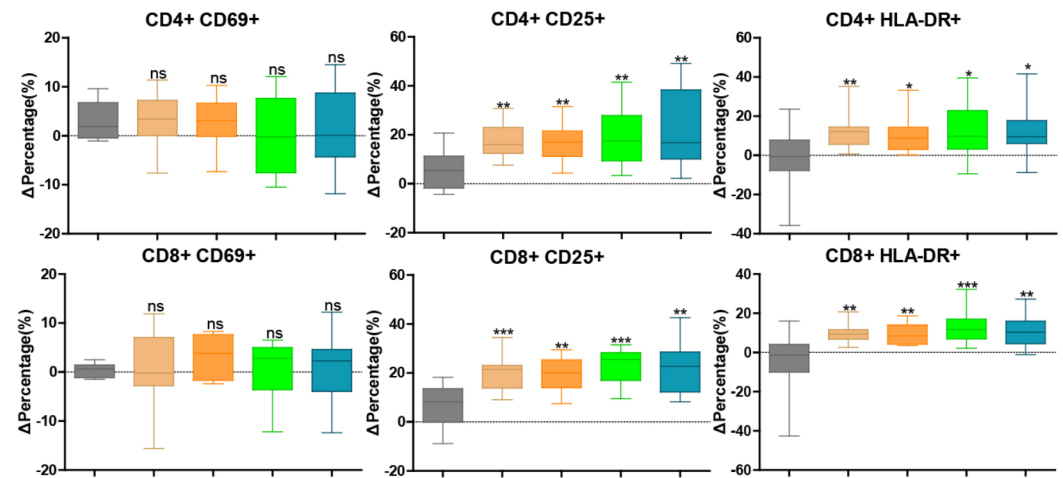

+SNT16

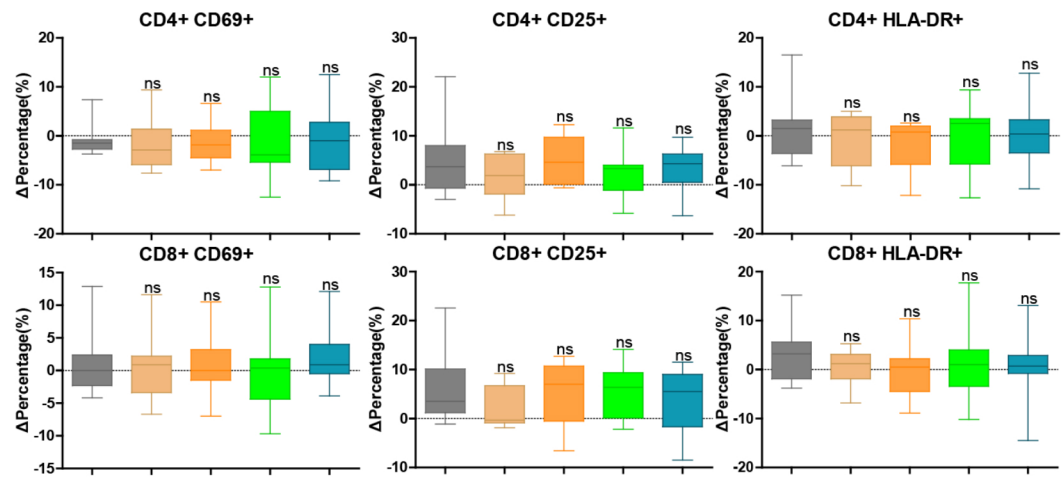

Supplement: Supplementary file 1 — Additional file 1: Fig. S1. A: CD38 and LMP1 expression in NKTCL patients. Fig. S2. CAR expression was detected by FITC-labeled human CD38 protein or EGFP. Fig. S3. Activation markers (CD69, CD25 and HLA-DR) expression on CD4+ and CD8+ subgroups of CAR-T cells co-cultured with YT, KAI3, SNK6 or SNT16. Fig. S4. Statistical results of activation markers expression on CAR-T cells co-cultured with YT, KAI3, SNK6 or SNT16. Fig. S5. Statistical results of cytokines releasing of CAR-T cells co-cultured with YT, KAI3, SNK6 or SNT16. Fig. S6. Flow histogram showed fluorescence intensity of each cytokine. Fig. S7. Survival curve data for each experimental group. [file 12916_2023_3040_MOESM1_ESM.zip › fig s4R5.pdf]

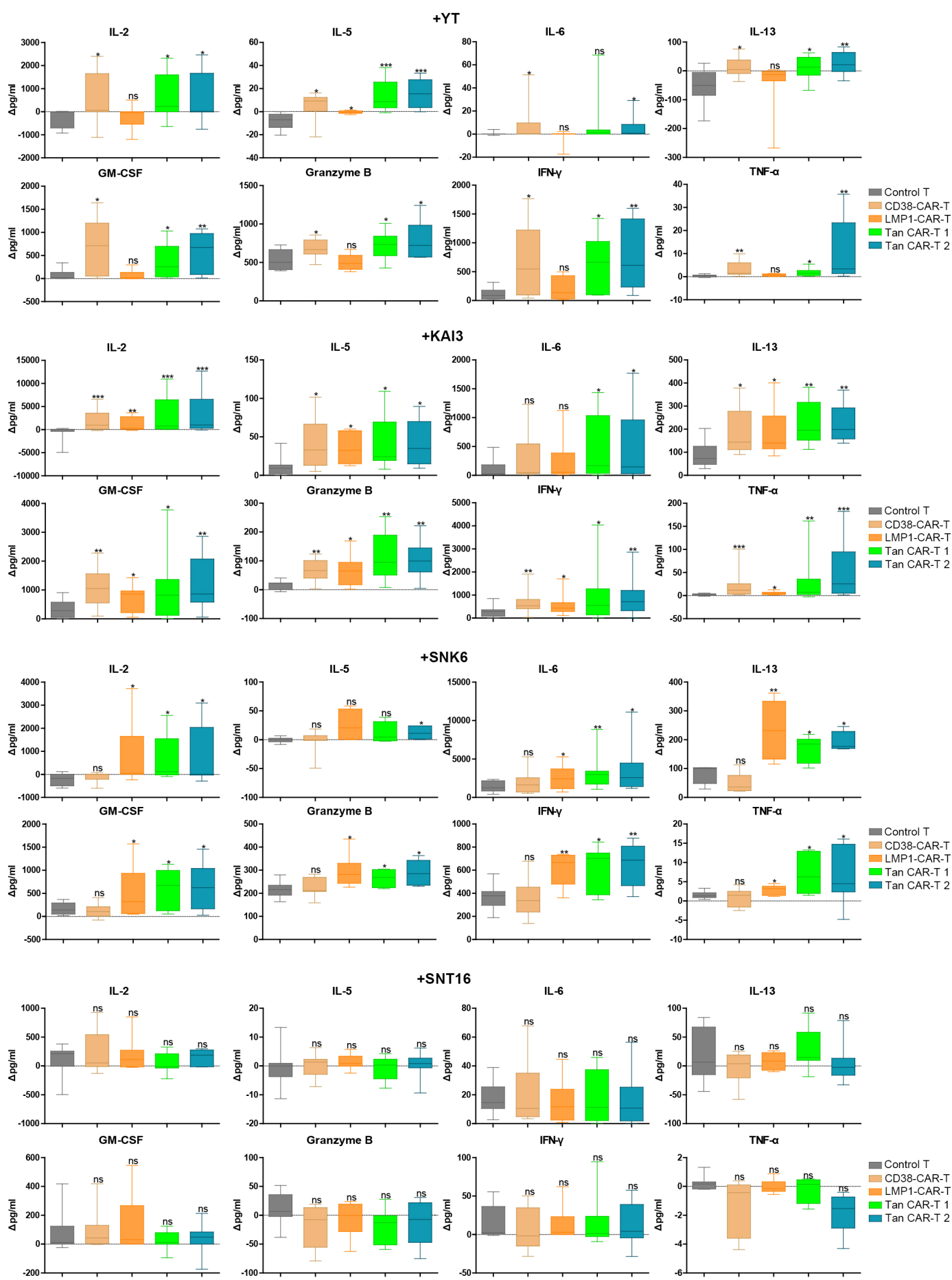

Supplement: Supplementary file 1 — Additional file 1: Fig. S1. A: CD38 and LMP1 expression in NKTCL patients. Fig. S2. CAR expression was detected by FITC-labeled human CD38 protein or EGFP. Fig. S3. Activation markers (CD69, CD25 and HLA-DR) expression on CD4+ and CD8+ subgroups of CAR-T cells co-cultured with YT, KAI3, SNK6 or SNT16. Fig. S4. Statistical results of activation markers expression on CAR-T cells co-cultured with YT, KAI3, SNK6 or SNT16. Fig. S5. Statistical results of cytokines releasing of CAR-T cells co-cultured with YT, KAI3, SNK6 or SNT16. Fig. S6. Flow histogram showed fluorescence intensity of each cytokine. Fig. S7. Survival curve data for each experimental group. [file 12916_2023_3040_MOESM1_ESM.zip › fig s5R5.pdf]

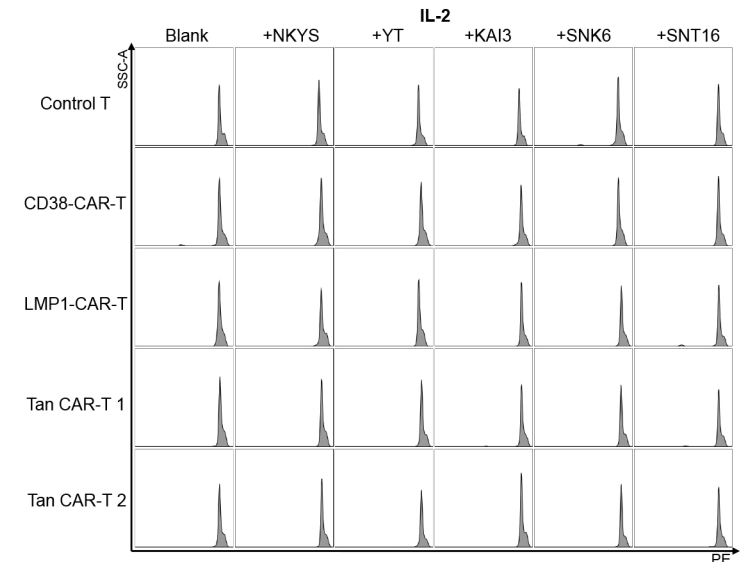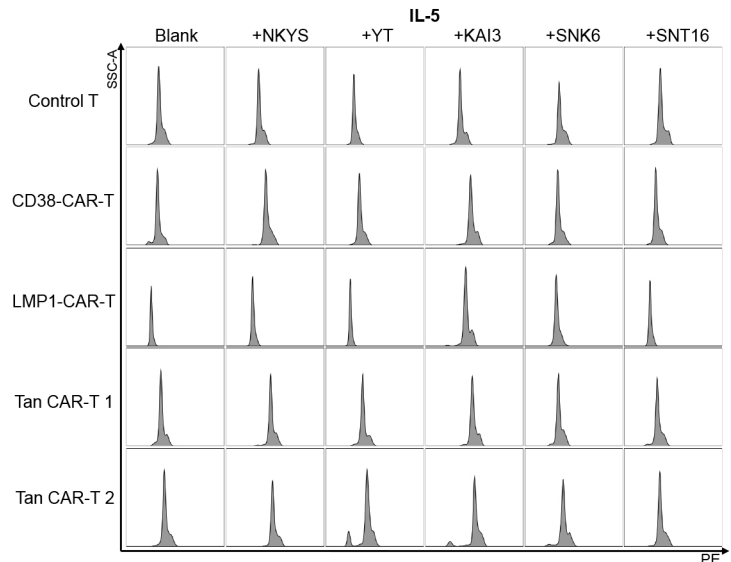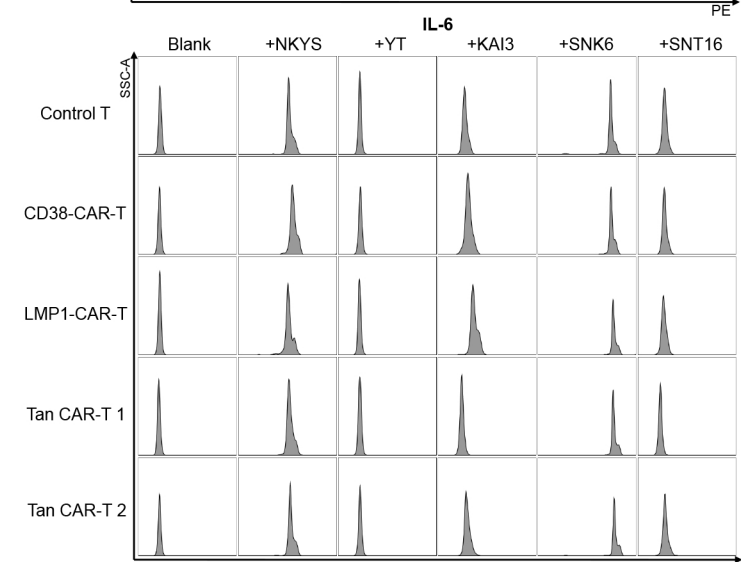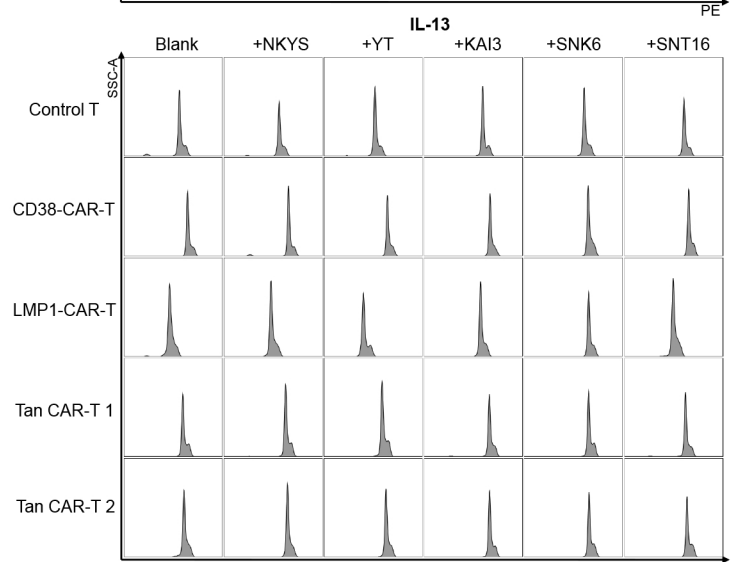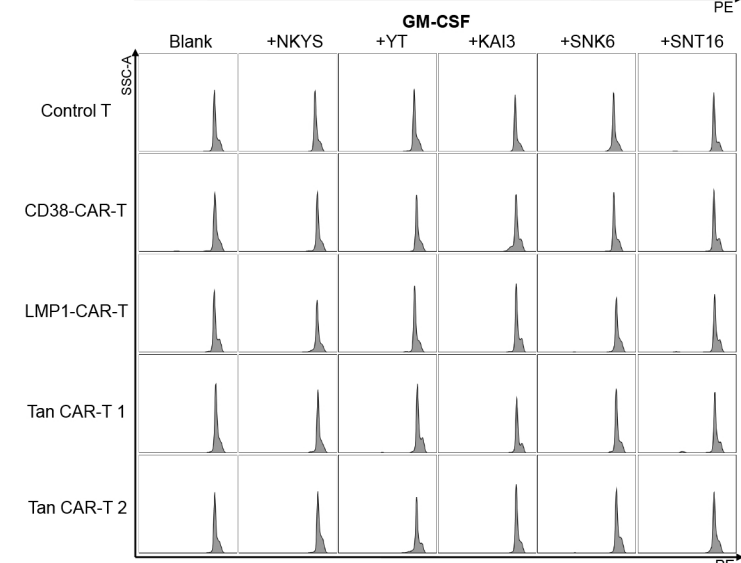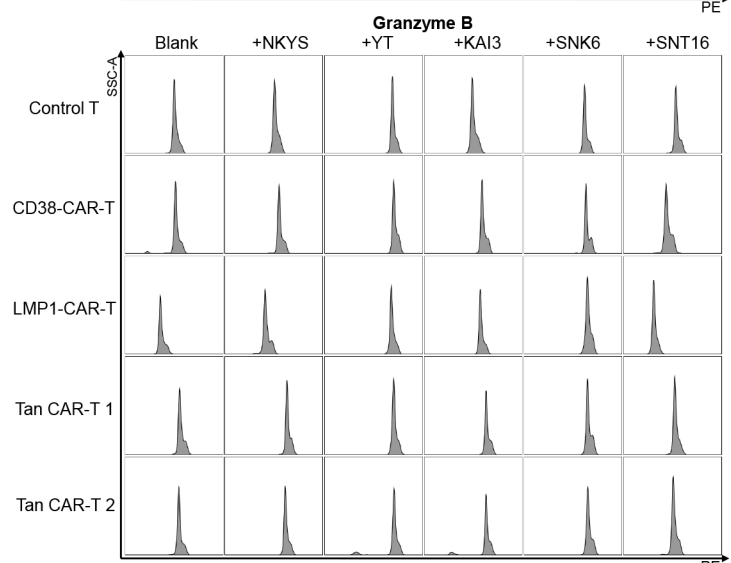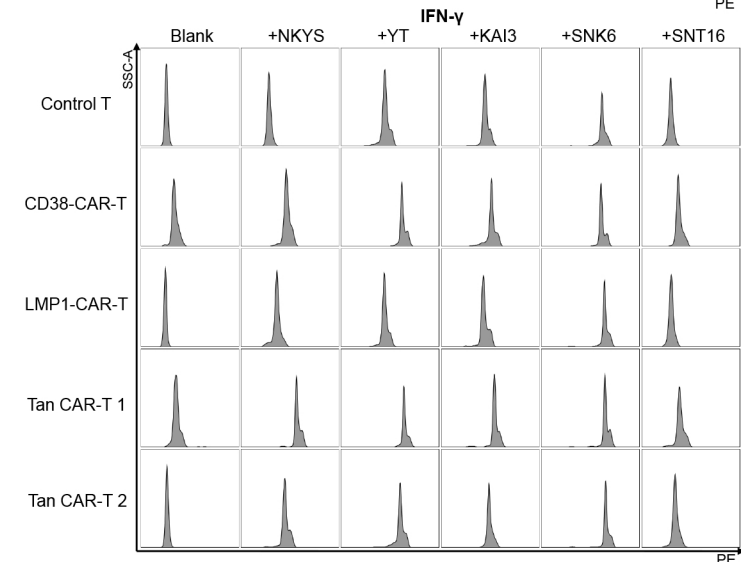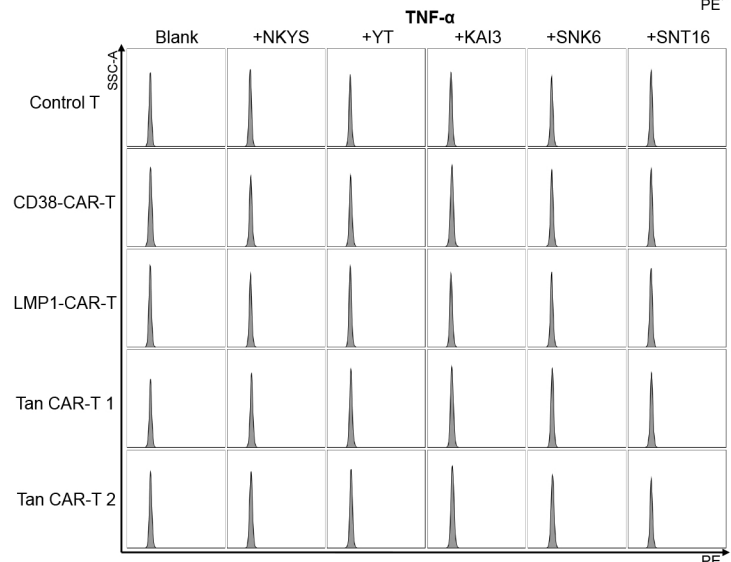

Supplement: Supplementary file 1 — Additional file 1: Fig. S1. A: CD38 and LMP1 expression in NKTCL patients. Fig. S2. CAR expression was detected by FITC-labeled human CD38 protein or EGFP. Fig. S3. Activation markers (CD69, CD25 and HLA-DR) expression on CD4+ and CD8+ subgroups of CAR-T cells co-cultured with YT, KAI3, SNK6 or SNT16. Fig. S4. Statistical results of activation markers expression on CAR-T cells co-cultured with YT, KAI3, SNK6 or SNT16. Fig. S5. Statistical results of cytokines releasing of CAR-T cells co-cultured with YT, KAI3, SNK6 or SNT16. Fig. S6. Flow histogram showed fluorescence intensity of each cytokine. Fig. S7. Survival curve data for each experimental group. [file 12916_2023_3040_MOESM1_ESM.zip › fig s6R5.pdf]

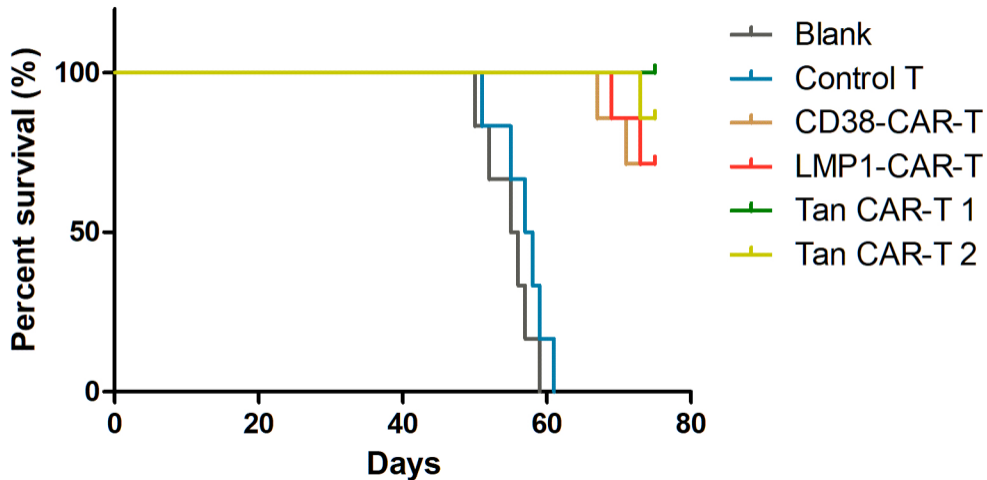

Supplement: Supplementary file 1 — Additional file 1: Fig. S1. A: CD38 and LMP1 expression in NKTCL patients. Fig. S2. CAR expression was detected by FITC-labeled human CD38 protein or EGFP. Fig. S3. Activation markers (CD69, CD25 and HLA-DR) expression on CD4+ and CD8+ subgroups of CAR-T cells co-cultured with YT, KAI3, SNK6 or SNT16. Fig. S4. Statistical results of activation markers expression on CAR-T cells co-cultured with YT, KAI3, SNK6 or SNT16. Fig. S5. Statistical results of cytokines releasing of CAR-T cells co-cultured with YT, KAI3, SNK6 or SNT16. Fig. S6. Flow histogram showed fluorescence intensity of each cytokine. Fig. S7. Survival curve data for each experimental group. [file 12916_2023_3040_MOESM1_ESM.zip › fig s7R5.pdf]
